# Heat shock protein 90: biological functions, diseases, and therapeutic targets

**DOI:** 10.1002/mco2.470

**Published:** 2024-01-25

**Authors:** Huiyun Wei, Yingying Zhang, Yilin Jia, Xunan Chen, Tengda Niu, Aniruddha Chatterjee, Pengxing He, Guiqin Hou

**Affiliations:** ^1^ State Key Laboratory of Esophageal Cancer Prevention & Treatment, Key Laboratory of Advanced Drug Preparation Technologies, Ministry of Education School of Pharmaceutical Sciences Zhengzhou University Zhengzhou China; ^2^ Department of Pathology Dunedin School of Medicine University of Otago Dunedin New Zealand

**Keywords:** client protein, co‐chaperone, disease, HSP90, inhibitor

## Abstract

Heat shock protein 90 (Hsp90) is a predominant member among Heat shock proteins (HSPs), playing a central role in cellular protection and maintenance by aiding in the folding, stabilization, and modification of diverse protein substrates. It collaborates with various co‐chaperones to manage ATPase‐driven conformational changes in its dimer during client protein processing. Hsp90 is critical in cellular function, supporting the proper operation of numerous proteins, many of which are linked to diseases such as cancer, Alzheimer's, neurodegenerative conditions, and infectious diseases. Recognizing the significance of these client proteins across diverse diseases, there is a growing interest in targeting Hsp90 and its co‐chaperones for potential therapeutic strategies. This review described biological background of HSPs and the structural characteristics of HSP90. Additionally, it discusses the regulatory role of heat shock factor‐1 (HSF‐1) in modulating HSP90 and sheds light on the dynamic chaperone cycle of HSP90. Furthermore, the review discusses the specific contributions of HSP90 in various disease contexts, especially in cancer. It also summarizes HSP90 inhibitors for cancer treatment, offering a thoughtful analysis of their strengths and limitations. These advancements in research expand our understanding of HSP90 and open up new avenues for considering HSP90 as a promising target for therapeutic intervention in a range of diseases.

## Graphical abstract

Targeting the dynamic cycling of HSP90 and its interaction with co‐chaperones can promote protein maturation and client protein degradation, potentially offering effective antineoplastic strategies.

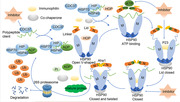

## 1 INTRODUCTION

Heat shock proteins (HSPs) represent a vast family of proteins present in both eukaryotic and bacterial organisms. The HSP family includes various members categorized based on their molecular weight, such as HSP70, HSP90, and HSP60. These proteins act as molecular chaperones, assisting in protein folding, preventing protein aggregation, and aiding in the repair of damaged proteins under stress conditions and their expression is induced in response to heat shock and other stressors.^1^, ^2^ Among the HSP family members, HSP90 holds significant importance. The chaperone mechanism of HSP90 is a critical regulator of proteostasis in eukaryotic cells, governing protein stability under both normal physiological conditions and stress‐induced scenarios. Studies have found that the expression of HSP90 in tumors is 2−10 times higher than in normal cells.^3^, ^4^, ^5^ Normally, HSP90 is highly abundant in both eukaryotic and bacterial cells. However, when expose to stress, HSP90 levels increase by approximately 4−6% compared with normal conditions.^6^, ^7^ HSP90 plays a crucial role in various essential cellular processes and regulates signaling pathways associated with cell proliferation, differentiation, migration, angiogenesis, protein folding, transportation, and degradation.^8^, ^9^ Through these functions, HSP90 facilitates the expression of genetic variants in its client proteins, thereby enhancing an organism's ability to adapt and survive in stressful environments.^10^ Moreover, highly abnormal HSP90 expression is associated with various pathological states, such as neurodegenerative disease, inflammation, aging‐related diseases and cancer.^11^ Especially, HSP90 exhibits high levels of expression across nearly all cancer types including lung, esophageal, gastric, breast, and colorectal cancer.^12^, ^13^ As a result, client proteins and inhibitors of HSP90 are a promising avenue for various diseases and cancer therapy. By targeting HSP90 and degrading its clients, multiple downstream signaling pathways can be effectively blocked, resulting in a reduction of the feedback activation effect and leading to more effective treatment.^14^ Therefore, HSP90 is a very promising target for the treatment of cancer and other diseases.

In this review, we begin by exploring the biological context of the HSPs. We then delve into the structural characteristics and biological functions of HSP90. Our discussion further examines the interplay between HSF‐1 and HSP90, along with the dynamic chaperone cycle of HSP90. With a focus on its relevance in various diseases, notably cancers, we outline the inhibitors used for HSP90 in cancer treatment, detailing both their benefits and limitations. Through this comprehensive analysis, we underscore the profound relationship between HSP90 and human health, pointing toward promising avenues for future medical advancements and treatments.

## 2 BIOLOGICAL BACKGROUND OF THE HSP FAMILY

In the early 1960s, Italian biologist Ferruccio Ritossa made a groundbreaking discovery, identifying HSPs.^15^, ^16^ He placed drosophila flies at temperatures exceeding their normal physiological range and observed that the chromosome ends of salivary glands were unexpectedly “puffing.” This puffing phenomenon was caused by the activation of regulatory factors, which induces the transcription of specific genes and overexpression of particular proteins in response to heat stress. These adaptive responses help cell survival under stress conditions.^17^ The proteins found to be overexpressed in these situations were identified as HSPs or stress proteins, which are a class of proteins that becomes notably more abundant when cells experience physical, chemical or mechanical damage or stress induced by factors such as high temperature, ischemia, hypoxia, oxidative stress, nutritional deficiency, inflammation, cancer, UV exposure, or trauma.^18^ This reaction is commonly referred to as the heat shock or heat stress response. HSPs are widely distributed throughout tissues and organs, facilitating the correct folding and maturation of numerous proteins, such as matrix metalloproteinase 9 (MMP9), mesenchychymal‐epithelial transition factor (Met), and human epermal growth factor receptor 2 (HER2), thereby helping cells in their adaptation to stressful environments. Consequently, they are a prominent target in the field of tumor therapy research.^13^, ^19^, ^20^

HSPs can be categorized into five distinct families: Small HSP, HSP60, HSP70, HSP90, and HSP110. These families differ in molecular weight and exhibit diverse structures and functions. Small HSPs play a role in triggering an immune‐regulatory response, prompting macrophages to counteract inflammation.^21^ HSP60, a typical mitochondrial molecular chaperone, assists nascent polypeptides in achieving their native conformation.^22^ HSP70 promotes cell survival, while HSP90 plays a multifaceted role in both neoplastic and normal cellular functions.^23^, ^24^ Additionally, overexpression of HSP110 in cultured mammalian cells increases thermal tolerance.^1^, ^25^ Among all the HSP family members, HSP90 holds a paramount role as a critical chaperone, orchestrating the folding and maturation of over 400 client proteins and stands out as one of the most crucial and versatile players in cellular processes,^26^ which makes it a central focus in scientific research and therapeutic development.

## 3 BIOLOGICAL FUNCTION AND STRUCTURE OF HSP90 COMPLEX

Eukaryotic cells contain four primary variants of HSP90, strategically distributed in various cellular compartments. HSP90α and HSP90β are predominantly located in the cytoplasm, while 94 kDa glucose‐associated regulatory protein (GRP94) is situated in the endoplasmic reticulum (ER), and tumor necrosis factor receptor‐associated protein 1 (TRAP1) resides within the mitochondrial stroma.^27^ Notably, HSP90α, also known as inducible HSP90, can be readily induced by heat or other stressors, whereas HSP90β is consistently expressed under normal conditions.^28^ The unique localization of each HSP90 subtype performs a distinct functional roles.^29^ Hsp90α promotes the chronic inflammation of cancer‐associated fibroblasts.^28^ Meanwhile, HSP90β plays a pivotal role in regulating lipid homeostasis, influencing the formation of endodermal progenitor cells, and promoting the cell migration and invasion in hepatocellular carcinoma.^30^, ^31^, ^32^ GRP94 is mainly associated with processes related to cell proliferation and metastasis in cancer.^33^ Finally, TRAP1 plays a vital role in metabolic regulation, dynamically adapting to shifting environmental conditions, and serving as a safeguard against potential harm.^34^ Following, we will embark on a comprehensive examination of the specific structural attributes and functional roles of these four discrete HSP90 subtypes.

### 3.1 HSP90

HSP90 functions as an ATP‐dependent molecular chaperone and exists in a homodimeric form, with options of HSP90αα or HSP90ββ; dimerization is necessary for its active chaperone function.^35^, ^36^ HSP90 comprises three distinct domains: the amino‐terminal domain (NTD), the intermediate domain (MD), and the carboxy‐terminal domain (CTD).^35^, ^37^, ^38^ The NTD and MD are connected by a flexible charged linker, playing a regulatory role in their interaction and enabling the rearrangement of domains during chaperone cycle of Hsp90.^39^ The NTD serves as the nucleotide‐binding site and specifically binds ATP in its pocket. It is also the primary target for nucleotide‐binding of HSP90 inhibitors.^9^ When ATP binds to the NTD of HSP90, it induces a conformational change and monomer distortion, converting the open V‐shaped conformation of HSP90 to a closed state.^40^ The client protein is wrapped between the two monomers, and as ATP hydrolysis occurs, the released energy drives the conformational cycle ultimately leading to release of the mature protein. The MD binds to client proteins and co‐chaperones and also serves as the binding site of protein kinase B (Akt).^39^ CTD, a nonspecific domain, can bind both adenine and pyrimidine nucleotides, in contrast to the NTD, and facilitates dimerization of HSP90. CTD contains a significant Met‐Glu‐Glu‐Val‐Asp motif, a crucial interaction site for chaperone molecules in the tetratricopeptide repeat domain.^41^ Furthermore, the second nucleotide‐binding site in CTD remains in a closed and inactive state until the ATP‐binding site in the N‐terminal domain (NTD) is engaged. Consequently, the CTD can regulate the activity of the N‐terminal ATPase.^42^ The distinctive structural features of HSP90 indicates its function flexibility, laying a foundation for research on HSP90‐specific inhibitors.

### 3.2 GRP94

GRP94 is highly conserved in mammals and primarily located in the ER.^43^ As the most prevalent glycoprotein in the ER, GRP94 is also recognized as an ER stress protein and shares a 50% homology with cytoplasmic HSP90.^44^ GRP94 has three primary conformations: Extended, partially extended and closed. The extended conformation primarily promotes binding of client protein and nucleotide to GRP94.^45^ Similar to HSP90, GRP94 experiences conformational changes when interacting with client proteins and co‐chaperones, transitioning from an open V‐shaped state to a closed state during this process. Furthermore, GRP94 consists of three primary domains: NTD, MD, and CTD, albeit with some distinctions when compared with HSP90.^9^ For example, the NTD end of GRP94 is longer than that of HSP90, suggesting conformational differences.^46^

GRP94 possesses a relatively shorter charged linker, enriched in lysine residues, resulting in a higher acidity. Furthermore, it is characterized by numerous calcium‐binding sites. Upon binding with Ca^2+^, GRP94 regulates its ability to bind polypeptides by promoting binding to the protein's N‐terminal region, which can affect GRP94's conformation and activity.^47^ The charged junction region of GRP94 regulates conformational changes during ATP hydrolysis; a deficiency in the linker may affect the activation of HSP90 ATPase and its ability to hydrolyze and bind client proteins.^48^

### 3.3 TRAP1

TRAP1 is primarily situated in the mitochondrial stroma, with a smaller portion fraction located in the outer membrane.^49^ In comparison with cytoplasmic HSP90, TRAP1 exhibits a higher degree homology.^50^ Structurally, TRAP1 is composed of NTD, MD and CTD domains, although it lacks a flexible charged linker, a feature present in HSP90. Notably, TRAP1 functions autonomously, without the assistance of co‐chaperones such as P23 and HSP70/90 organizing protein (HOP).^49^ TRAP1's affinity for ATP is up to 10 times greater than that of HSP90, and it displays heightened sensitivity to HSP90 inhibitors such as geldanamycin (GM) and radicicol.^9^, ^51^, ^52^ Additionally, TRAP1 can be activated by heat stress, with its expression increasing over 200‐fold under elevated temperature conditions.^53^

In summary, HSP90 is a versatile molecular chaperone that relies on ATP for assisting in protein folding, with each HSP90 subtype playing a distinct biological role. These subtypes are integral to a range of physiological and pathological processes by maintaining protein homeostasis in response to stress.^29^ It is noteworthy that the heat shock factor‐1 (HSF‐1) serves as the central regulator, intricately connecting with HSP90 to regulate a sound of signaling proteins expression and activity. HSP90 and HSF1 orchestrates the cellular response to stressors, ensuring the cell's ability to adapt and thrive in challenging conditions.^54^

## 4 CROSS‐TALK BETWEEN HSF‐1 AND HSP90

HSF1 is a highly conserved transcription factor, exhibits widespread expression in various eukaryotic organisms. It serves as the master regulator of the heat shock response, controlling the transcription of HSPs.^55^ In ordinary physiological circumstances, HSF‐1 primarily associates with HSP90, alongside co‐chaperones P23 and FK506‐binding protein 52 (FKBP52), maintaining an inactive state.^56^, ^57^, ^58^ Nevertheless, when exposed to stress‐inducing circumstances, such as burns or high fever, HSF‐1 protein dissociates from its complex and forms homotrimers, which subsequently translocate to the nucleus. Inside the nucleus, these HSF‐1 homotrimers bind to heat shock transcription elements (HSEs) and transform into active trimers. Subsequently, they interact with the HSP90 promoter region, obtaining specific DNA‐binding capabilities and transcription‐enhancing activities. This leads to an increase in the transcription of HSP90, HSP70, and other co‐chaperones, while concurrently suppressing the transcription of other genes.^59^, ^60^, ^61^, ^62^ This mechanism plays a pivotal role in maintaining stable and elevated HSP90 expression under stress conditions. Once a sufficient quantity of HSP90 proteins is synthesized, they exert inhibitory effects on HSF‐1 expression, contributing to protein homeostasis through a feedback loop. These molecular chaperones also have substantial implications for the expression of oncogenes like Met, epidermal growth factor receptor (EGFR), RAF, and Akt.^63^, ^64^

**FIGURE 1:**
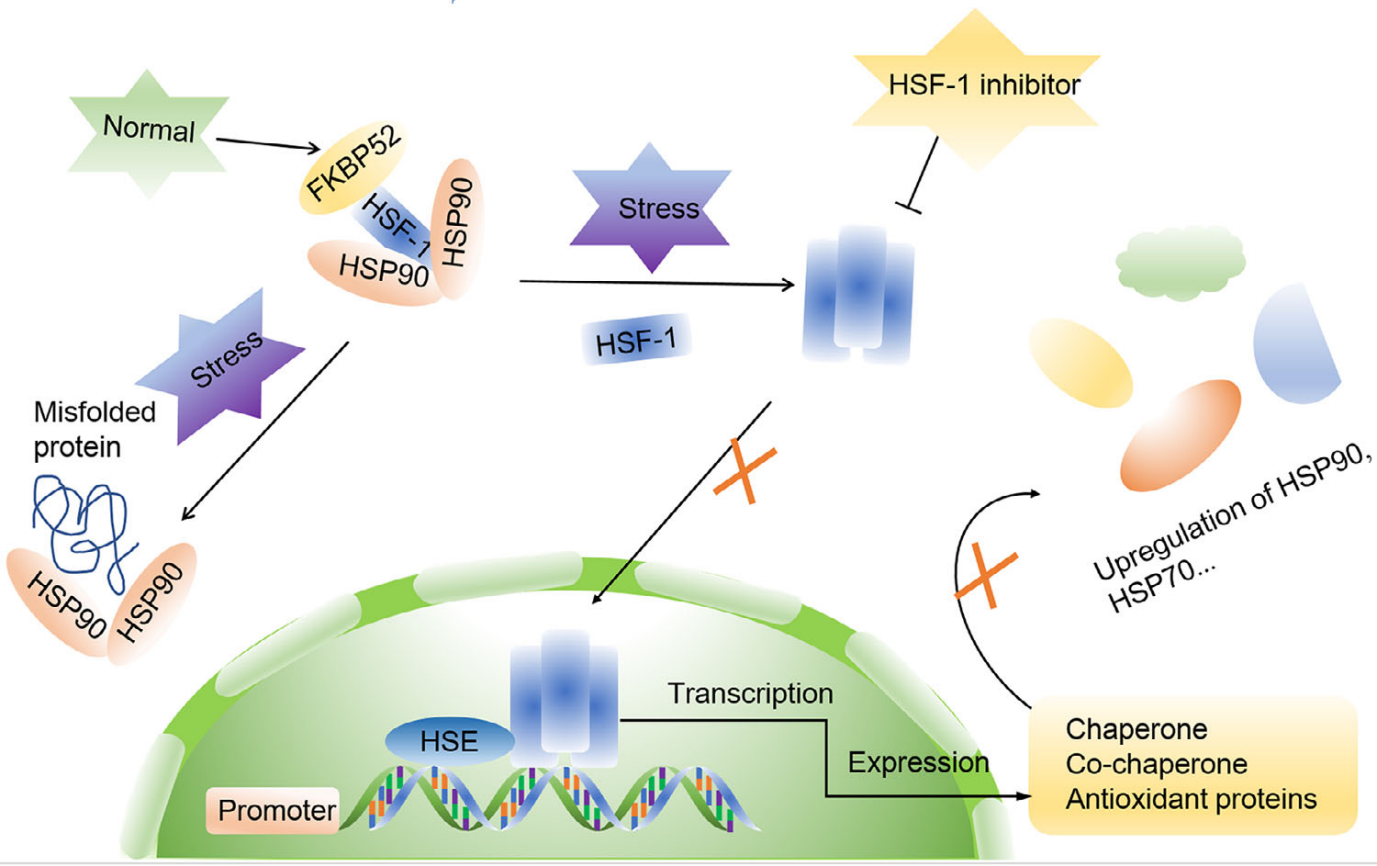
Interaction between HSP90 and HSF‐1. In unstressed conditions, HSF‐1 remains in an inactive state by forming a complex with HSP90 and FKBP52. However, under stress, HSF‐1 is released from this complex, leading to the formation of active homotrimers. These active HSF‐1 homotrimers translocate to the nucleus, where they initiate the transcription of HSPs. HSF1 inhibitors can prevent its nuclear translocation, thus modulating its activity. HSP, heat shock protein; HSF‐1, heat shock factor‐1; FKBP52, FK506‐binding protein 52; HSE, heat shock transcription element.

Studies have shown that elevated expression of HSF‐1 in tumor cells promotes the initiation and progression of tumors by stimulating transcription of oncogenes, regulating tumor cell proliferation and anabolism, and contributing to evasion of apoptosis.^65^, ^66^, ^67^ Consequently, HSF‐1 has garnered significant attention as a promising therapeutic target for various cancer types,^66^, ^68^, ^69^, ^70^ with HSF‐1 inhibitors holding promise as potential antitumor agents, increasing the efficacy of HSP90 inhibitors (Figure 1).

Apart from targeting HSF‐1, another promising avenue in cancer treatment is focusing on the dynamic chaperone cycle of HSP90. This approach has garnered growing interest in recent years, primarily driven by the recognition that the HSP90 chaperone mechanism plays a crucial role in maintaining protein homeostasis within eukaryotic cells, both under normal physiological conditions and during times of stress.^71^

## 5 HSP90 DYNAMIC CHAPERONE CYCLE

**FIGURE 2:**
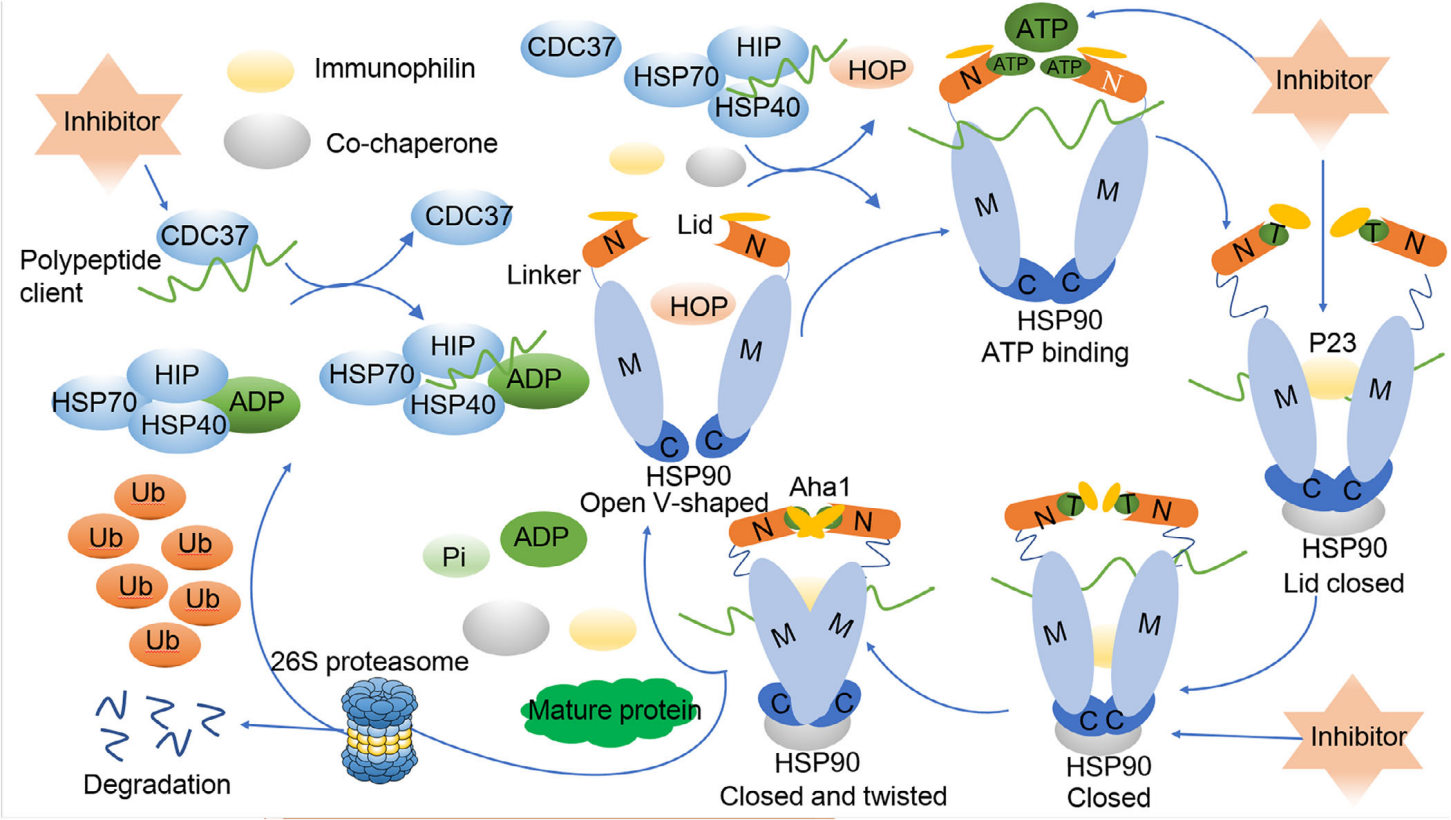
HSP90 chaperone cycle. HSP90 chaperone undergoes dynamic cycling process, transitioning between open and closed states to promote maturation of polypeptide clients into functional proteins with the help of ATP and a variety of co‐chaperones. Targeting Hsp90 and its interaction with co‐chaperone could lead to the degradation of client proteins by 26S proteasome. HSP, heat shock protein; CDC37, co‐chaperone cell division cycle 37; HIP, the Hsp70‐interacting protein; Ub, ubiquitin; Pi, pyrophosphoric acid; N, NTD; M, MD; C, CTD; HOP, The Hsp70/Hsp90 organizing protein; Aha1, activator of heat shock 90 kDa protein ATPase homolog 1.

Within eukaryotic organisms, HSP90 plays a vital role as a molecular chaperone, guiding client proteins through their intricate folding process. This function ensures the stability of these proteins and facilitates accurate assembly of protein complexes, ultimately promoting their maturation. Furthermore, HSP90 contributes to reducing the accumulation of harmful stress‐induced proteins by facilitating their degradation through co‐chaperones such as activator of heat shock 90 kDa protein ATPase homolog 1 (AHA1), HOP, co‐chaperone cell division cycle 37 (CDC37), and P23. These co‐chaperones aid in directing the client proteins to undergo degradation via either the ubiquitin/proteasome or lysosome pathways (Figure 2).^72^, ^73^, ^74^, ^75^

The HSP90 chaperone is intricately involved in a dynamic cycling process, continuously transitioning between open and closed states. Extensive studies focusing on steroid receptors in yeast cells have delved into the intricacies of this HSP90 chaperone cycle.^76^, ^77^ In the open state, the two N‐termini of the HSP90 monomer are spatially separated, and with the assistance of co‐chaperones, they bind to client proteins in an unbound state. An example of this process is CDC37, which plays a pivotal role in recruiting polypeptide clients, including newly synthesized natural peptides or misfolded steroid hormone receptors.^78^ Subsequently, these client proteins interact with the HSP70/HSP40 complex, safeguarding them against potential aggregation. The stability of protein complex's is further enhanced by HSP70‐interacting protein (HIP), which promotes the conversion of ADP to ATP.^79^

HSP90 initiates its action by binding to client proteins, which are wrapped by the HSP70/HSP40 protein complex. The interaction between HSP90 and HSP70 is facilitated by the adaptor protein HOP, which simultaneously binds to both HSP90 and HSP70.^80^ The introduction of immunophilins such as FKBP51 and FKBP52 to the HSP90 homodimer results in the formation of an active heteroprotein complex, leading to the simultaneous release of HSP70, HIP, and HOP.^81^ Upon the binding of ATP to the NTD of HSP90 in the heteroprotein complex, HSP90 undergoes a transition from an open to a closed state. However, HSP90 inhibitors can disrupt this process by competing with ATP for the NTD binding site, ultimately leading to protein degradation by the proteasome.^82^ In the absence of inhibitors disrupting the folding cycle, other co‐chaperones like P23 and AHA1 become actively engaged. AHA1 binds to the MD of HSP90, stimulating ATP hydrolysis. This interaction supports the folding of the bound client proteins and enhances the release of immunophilins and co‐chaperones.^83^, ^84^ Finally, ATP is hydrolyzed, resulting in the release of ADP, pyrophosphoric acid, any remaining co‐chaperones, and the fully matured client proteins. HSP90 then reverts to its open conformation, ready for another cycle.^71^, ^85^

## 6 THE RELATIONSHIP BETWEEN HSP90 AND VARIOUS DISEASES

The attainment of a well‐defined three‐dimensional structure is essential for proteins to function effectively within cells. Unfortunately, protein misfolding and subsequent aggregate formation frequently underlie various diseases, including cancer and neurodegenerative disorders.^11^ The misfolding and aggregation of specific proteins have been linked to the actions of HSP90 chaperone machinery,^86^ implicating HSP90 chaperone and its regulation in the pathogenesis of these diseases, and rendering it as a potential target for therapeutic interventions in several diseases.

### 6.1 HSP90 in neurodegenerative diseases

A recent study utilizing a mouse model of Alzheimer's disease (AD) has presented compelling evidence that the presence of insoluble tau in neuronal cells disrupts the solubility of numerous other proteins, resulting in a systemic breakdown of cellular homeostasis.^87^ In AD patients, the characteristic Tau tangles and β‐amyloid deposits colocalize with HSP90, and HSP90 plays a pivotal role in the regulation of their aggregation and degradation.^88^ Intriguingly, work form Chen et al.^89^ has suggested that the neurotoxicity induced by β‐amyloid can be mitigated through the use of the HSP90 inhibitor, 17‐AAG, which in turn contributes to the restoration of synaptic function. Moreover, FKBP51, in collaboration with HSP90, exerts control over glucocorticoid receptor activity through a brief negative feedback loop. This intricate regulatory mechanism holds implications for psychiatric disorders like depression.^56^, ^90^ Beyond AD, HSP90, along with its co‐chaperones, also plays a role in the regulation of Huntington's and Parkinson's diseases.^91^

In summary, the HSP90 chaperone machinery plays a widespread role in the pathogenesis and potential therapeutic approaches for various neurodegenerative diseases. It achieves this by orchestrating the balance between HSP90 and different co‐chaperones, with the Hsp90/Cdc37 complex emerging as a potential target for the regulation of proteins associated with neurodegenerative conditions.^91^

### 6.2 HSP90 in cerebro‐cardiovascular diseases

HSP90 plays a pivotal role in facilitating the maturation of inducible NO synthase protein by engaging with the apoenzyme within cells and subsequently orchestrating heme insertion in an ATP‐dependent manner.^92^ Notably, Aceros et al.^93^ were the first to demonstrate that celastrol activates prosurvival signaling pathways, upregulating cytoprotective HSF1 and HO‐1, which are responsible for enhancing cardiac cell survival, particularly under hypoxic conditions. Further contributing to the realm of cardiac health, research led by Zhang X's team^94^ revealed that inhibiting S‐nitrosylation (SNO)‐HSP90 effectively mitigates fibrosis by disrupting the transforming growth factor‐β (TGF‐β)/SMAD3 signaling pathway, presenting a promising avenue for potential therapies aimed at addressing cardiac remodeling.

It was also found that the HSP90 inhibitor GA has the capacity to activate HSF1, resulting in heightened expression of Hsp70 and Hsp25, which in turn leads to a reduction in brain infarct volume and improved post‐ischemic behavioral outcomes.^95^ Meanwhile, Qi et al.^96^ proposed that 17‐DMAG not only reduces infarction but also ameliorates neurological deficits, while simultaneously inhibiting blood–brain barrier (BBB) disruption by downregulating MMP9. Additionally, 17‐DMAG at a dosage of 5 mg/kg was shown to curtail hematoma expansion effectively and contributed to improved neurological outcomes.^97^

In summary, the utilization of HSP90 inhibitors in conditions associated with BBB dysfunction holds promising potential for novel therapeutic interventions aimed at benefiting affected populations.^98^

### 6.3 HSP90 in infectious diseases

The cytosolic P. falciparum Hsp90 (PfHsp90) is a pivotal player in the development of the parasite, particularly during its intra‐erythrocytic stage within the human host.^99^ Protozoans, including Leishmania donovani and P. falciparum, the culprits behind leishmaniasis and malaria respectively, rely on HSP90 to navigate the temperature and pH variations they encounter throughout their life cycles, making the HSP90 system crucial for differentiation and development.^100^, ^101^ Consequently, the use of species‐specific inhibitors targeting parasite HSP90 presents a promising approach to impede their proliferation.^100^

Recent studies underscore the significant role of HSP90 in viral infections.^102^, ^103^ The demand for chaperones in viral infections is apparent, as viral proteins exhibit rapid translation rates, multifunctionality, and a need for conformational flexibility and proteolytic processing. For instance, duck hepatitis B virus’ reverse transcriptase relies on HSP90, alongside other chaperones such as HSP70/HSP40 and co‐chaperones like HOP and p23, acting as substrate release factors and supporting the incorporation of pre‐genomic RNA (p‐gRNA) into nucleocapsids.^104^, ^105^, ^106^ Additionally, the high mutation rate of viruses can result in the accumulation of potentially unstable proteins, and HSP90 serves as a buffer to these unstable proteins, aligning with its role in phenotypic evolution.^71^ Given these attributes, virus‐infected cells exhibit heightened sensitivity to HSP90 inhibitors compared with uninfected cells, offering a promising avenue for addressing virus‐related diseases.

### 6.4 HSP90 in aging diseases

Aging stands as the principal risk factor for numerous chronic degenerative diseases and as for cancer, with the accumulation of senescent cells in various tissues significantly contributing to the aging process and age‐related maladies.^107^ Kim et al.^108^ shed light on a fascinating positive feedback loop: the suppression of HSF1 activates the p38–NF‐κB–SASP pathway, subsequently propelling senescence. Conversely, overexpressing HSF1 inhibits the p38–NF‐κB–SASP pathway and partially alleviates senescence. Thus, the downregulation of HSF1 plays a pivotal role in either inducing or sustaining DNA damage signaling‐induced cell senescence.^108^ Recently, HSP90 inhibitors have been tested in multiple mouse models of aging to determine their potential to extend healthy lifespan, mitigate frailty, and enhance stem cell functionality. Notably, 17‐DMAG has demonstrated impressive senolytic effects, contributing to the enhancement of mouse health and longevity.^109^ Furthermore, an independent study revealed that in senescent cells, the application of 17‐DMAG (100 nM) led to a reduction in the levels of p‐AKT while leaving the levels of AKT, a client of Hsp90, Hsp90, and actin unaffected. This finding suggests that 17‐DMAG disrupts the interaction between AKT and Hsp90, preventing AKT phosphorylation.^107^ Conversely, GA has exhibited a contrasting behavior, inducing senescence at lower concentrations (0.1 μM) and demonstrating senolytic effects at higher concentrations (1 μM).^109^ Consequently, the role of Hsp90 in senescence is intricate and remains incompletely understood. The fundamental question lingers: Does Hsp90 inhibit aging? The answer appears to be both affirmative and negative. Hsp90 antagonists exhibit properties that can either promote or hinder the aging process, a distinction primarily dictated by the concentration of inhibitor molecules and the cellular environment.^110^

### 6.5 HSP90 in cancer

Tumor cells find themselves under constant stress due to the presence of mutant proteins and their rapid proliferation, which exerts additional pressure on the regulation of protein homeostasis. The intricate and dynamic nature of the acidic tumor metabolic microenvironment further exacerbates these proteostatic challenges. HSP90, functioning as a molecular chaperone, assumes a critical role in promoting the survival of cancer cells, largely because of the substantial reliance of these cells on HSP90‐assisted signaling pathways.^71^ Beyond the well‐known HSP90 clients like the tumor suppressor p53 and the oncoprotein SRC, a myriad of other HSP90 clients, including protein kinases, telomerase, hypoxia‐inducible factor 1α (HIF‐1α), and Akt, are deeply entrenched in the processes that fuel tumor growth.^23^, ^111^, ^112^, ^113^ It is worth noting that tumors exhibit a significant upregulation of HSP90 levels, and heightened HSP90 expression in breast cancer, for instance, has been linked to reduced patient survival rates.^114^ Consequently, HSP90 stands as a pivotal enabler of oncogene addiction and a key player in facilitating the survival of cancer cells, and inhibition of HSP90 can lead to the degradation of these client proteins, making it a target for anticancer therapies.^115^

Cancer represents a significant global health challenge. The GLOBOCAN 2020 report from the International Agency for Research on Cancer revealed that in 2020 alone, about 19.3 million new cancer cases emerged, resulting in close to 10.0 million deaths globally.^116^ Projections for 2040 are even more concerning, anticipating a rise in cancer cases to 28.4 million, marking a 47% increase from 2020.^116^ Such data underscore the imperative to delve deeper into the intricate molecular mechanisms of tumors and pinpoint potential therapeutic interventions.^117^ In this context, the exploration of HSP90 and its inhibitors emerges as a pivotal avenue for advancing cancer treatment strategies.

## 7 HSP90 AS A PROMISING TARGET FOR CANCER THERAPY

HSP90 exerts a substantial influence in various tumors and is closely associated with the pathological progression of carcinoma.^118^, ^119^, ^120^ Therefore, understanding the intricate relationship between HSP90 and cancer holds the promise of uncovering pivotal insights that could lead to the development of more rational and effective cancer treatment strategies.

### 7.1 Roles of HSP90 and its co‐chaperones in cancer

The chaperone function of HSP90 is intricately reliant on the coordination of co‐chaperones, which play a crucial role in supporting HSP90's unique function and maintaining cellular homeostasis. In tumor cells, where HSP90 is typically activated, oncogenes are heavily dependent on HSP90 to maintain their stability via chaperone circulation. Moreover, some overexpressed or mutated kinases, as well as oncogenic transcription factors, interact with HSP90 to drive tumor progression. HSP90 acts as a “double‐edged sword” that can be beneficial or harmful to human biology.^5^ In normal cells, the affinity between client proteins and HSP90 is relatively low, but HSP90 could still be beneficial to maintain the quality and stability of these client proteins.^121^, ^122^, ^123^ By orchestrating the formation of a super multimolecular chaperone complex, HSP90 stabilizes the conformation of client proteins, interrupting their degradation and activating crucial pathways such as PI3K/Akt/mTOR and mitogen‐acivated protein kinase (MAPK), which are pivotal in regulating tumor cell proliferation and survival. Furthermore, HSP90 acts as a facilitator to accelerate degradation of abnormal aggregation proteins and molecules proteins, thus contributing to the maintenance of cellular homeostasis. However, in the context of tumors and their interactions with co‐chaperones such as HSP70, HOP, HSP40, and P23, HSP90 exhibits heightened ATPase activity, promoting the maturation of carcinogenic client proteins.

**FIGURE 3:**
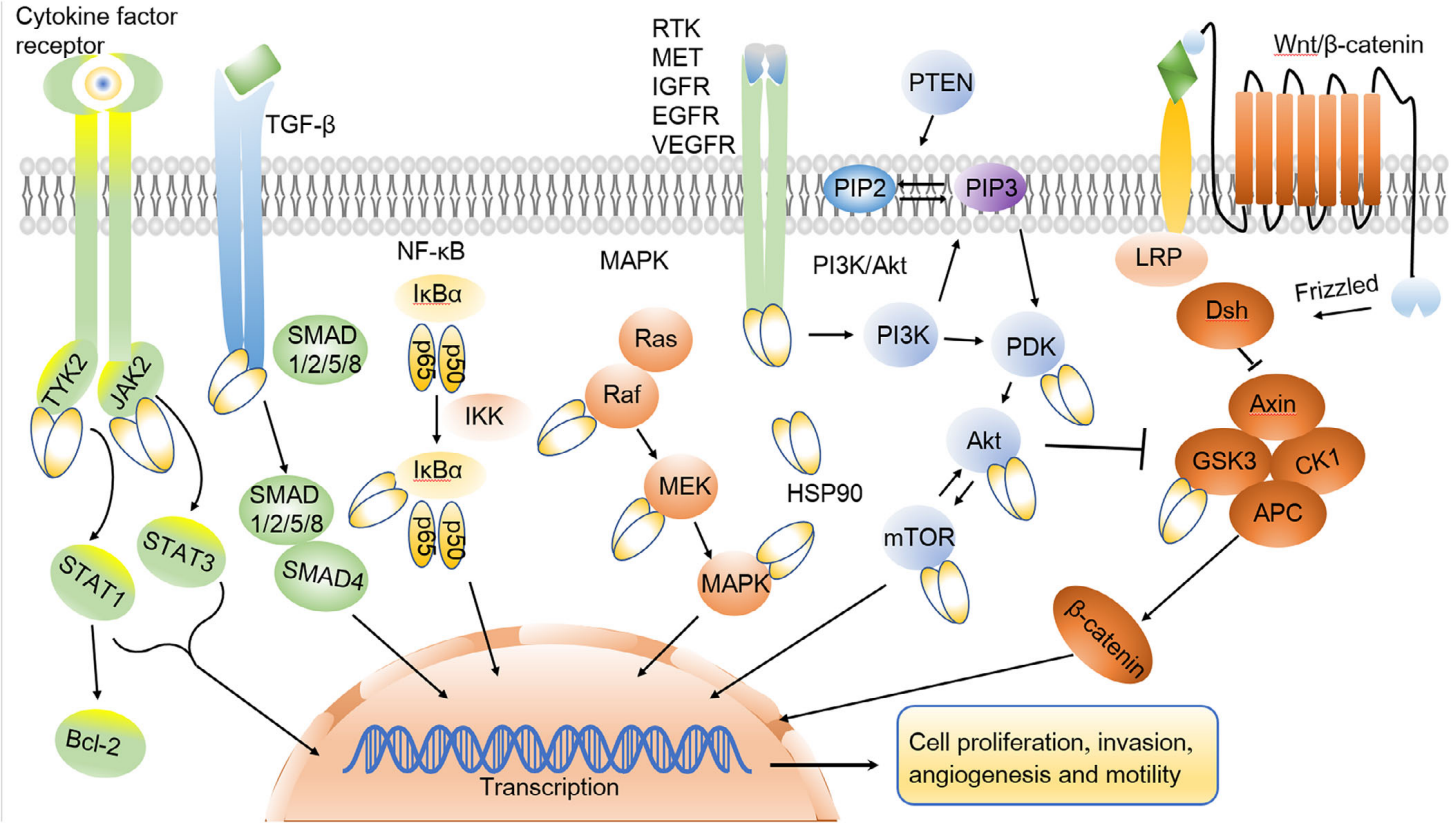
HSP90, clients and typical events in cancer. HSP90 plays a central role in six major oncogenic pathways involving client proteins, including the JAK/STAT, SMAD, NF‐κB, MAPK, PI3K/Akt, and Wnt/β‐catenin signaling pathways. These pathways collectively drive processes such as cell proliferation, invasion, angiogenesis, and motility in cancer. HSP, heat shock protein; TYK, tyrosine kinase; IKK, IkappaB kinase; RTK, receptor tyrosine kinase; IGFR, insulin/IGF‐1 transmembrane receptor; PIP2, phosphatidylinositol 4,5‐bisphosphate; PIP3, phosphatidylinositol 3,4,5‐trisphosphate; PDK, pyruvate dehydrogenase kinase; LRP, low‐density lipoprotein (LDL)‐related protein; Dsh, dishevelled protein; GSK3, glycogen synthase kinase 3; CK1, casein kinase 1; APC, adenomatosis polyposis coli.

A current compilation of HSP90 clients and interactors is available on Didier Picard's website at https://www.picard.ch/downloads/HSP90interactors.pdf.^124^ To date, a roster of over 400 known HSP90 client proteins has been assembled, most of which are oncogene products or key regulators of signal transduction pathways, significantly promoting the initiation and progression of cancer.^125^ Furthermore, the progression of cancer heavily relies on essential signaling pathways, each prominently featuring key oncoproteins recognized as HSP90 clients (Figure 3). For instance, the signaling cascade of TGF‐β plays a critical role in governing various cellular processes, including cell proliferation, differentiation, apoptosis, and migration.^126^ Notably, Met, a receptor for hepatocyte growth factor, experiences substantial upregulation in non‐small cell lung cancer (NSCLC) and gastric cancer, facilitating tumor invasion and participating in angiogenesis.^127^, ^128^ Similarly, the vascular endothelial growth factor (VEGF) receptor promotes angiogenesis, providing nutrition for the survival of breast cancer cells.^129^ Meanwhile, janus kinase (JAK2) and tyrosine kinase 2 (TYK2) possess the capability to induce the phosphorylation and activation of signl transducerand activator of transcription 1 (STAT1 )and STAT3, with the JAK2/tyrosine kinase 2 (TYK2)/STAT‐1/3 signaling axis being closely associated with tumor promotion.^130^, ^131^

Additionally, HSP90 plays a pivotal role in each of the three critical stages of tumor invasion: the degradation of the extracellular matrix, cell adhesion, and invasion into new sites. HSP90 is responsible for activating MMP2, significantly contributing to tumor migration and invasion.^132^, ^133^, ^134^ Other HSP90 clients include IκB kinases that regulate NF‐κB activation,^135^ and glycogen synthase kinase 3 (GSK3), regulated by the Wnt pathway and promoting cancer progression.^136^ When HSP90 binds to the prosurvival kinase Akt, it hinders proteasome‐mediated degradation of Akt, thereby reinforcing the functional stability of PI3K/Akt signaling and promoting cell survival.^12^, ^137^

HSP90 client proteins are classified into several categories, including protein kinase Met, human epidermal growth factor receptor‐2 (HER‐2) and EGFR, transcriptional subtype like Akt and mutant p53, cell cycle regulators including cyclin D and CDK4, HIF‐1α, apoptosis‐related such as Bcl‐2 and BAX, and structural proteins comprising microtubules and microfilaments.^138^, ^139^, ^140^, ^141^, ^142^, ^143^, ^144^ In terms of affinity, some client proteins and HSP90 exhibit a particular order, with HER2, mutant EGFR, Raf‐1, Akt, mutant v‐raf murine sarcoma viral oncogene homolog B1 and wild‐type EGFR showing preference for HSP90 interaction.^145^ These proteins, prone to conformational changes, are heavily dependent on HSP90 to maintain their functionality.^23^

As the activation and overexpression of HSP90 in various cancers including lung cancer, breast cancer, and colorectal cancer, researchers have aimed to target HSP90 to interfere with the interaction with client proteins., This disruption results in the irreversible degradation of client proteins by the proteasome or lysosome pathway.^23^, ^146^, ^147^ This strategy effectively blocks the survival pathway in tumor tissues or cells. Consequently, inhibiting HSP90 represents an attractive and selective strategy for effective treatment of cancer.^82^

### 7.2 Association between HSP90 and tumor resistance

Considering the malignant characteristics of tumors, particularly their propensity to metastasize and resistance to treatment, combating resistance is a significant challenge.^148^ Resistance often arises from genetic mutation or epigenetic changes triggered by prolonged exposure to chemotherapeutic agents. These changes activate alternate signaling pathways and lead to the overexpression of corresponding protein kinases or transcription factors, resulting in multidrug resistance.^149^ This resistance mechanism is closely related to the chaperone function of HSP90, as most of these proteins are HSP90 clients. For example, in the treatment of NSCLC with the EGFR‐targeting inhibitor gefitinib, the initial response is considerable. However, prolonged drug use can significantly decrease the antitumor effect due to the mutation of EGFR or the activation of Met, which in turn triggers downstream RAS/RAF/MEK/ERK and ERBB3/PI3K/Akt signaling pathways, promoting tumor proliferation and reducing sensitivity to gefitinib.^150^, ^151^, ^152^, ^153^ However, combination therapy with second‐generation HSP90 inhibitors such as STA9090 effectively counteracts resistance to gefitinib in NSCLC treatment.^154^ Similarly, resistance to the second‐generation anaplastic lymphoma kinase (ALK) inhibitor TAE684 in human neuroblastoma often results from mutations in ALK F1174L.^155^, ^156^, ^157^ Nonetheless, the HSP90 inhibitor AUY‐922 can hinder multiple signal transduction pathways by facilitating the degradation of various HSP90 client proteins, including Akt, ERK, and STAT3. This action effectively obstructs cell cycle progression and proves to be an effective means for overcoming resistance to ALK inhibitors in human neuroblastoma. Consequently, combination therapy with HSP90 inhibitors and targeted drugs can effectively inhibit multiple carcinogenic signaling pathways, enhancing the antitumor effects of targeted drugs. By targeting the active sites of HSP90, inhibitors of HSP90 inhibit its chaperone activity, interrupt multiple signaling pathways involved in tumor progression, cut off the tumor cell nutrition supply, block collateral circulation, and reduce tumor resistance caused by targeting a single factor. Therefore, targeting HSP90 is a promising and effective strategy for addressing cancer resistance,^115^, ^158^, ^159^, ^160^ and various HSP90 inhibitors have been developed and are currently undergoing evaluation in preclinical and clinical settings.^23^, ^29^, ^161^, ^162^, ^163^, ^164^

## 8 HSP90 INHIBITORS FOR CANCER TREATMENT

**FIGURE 4:**
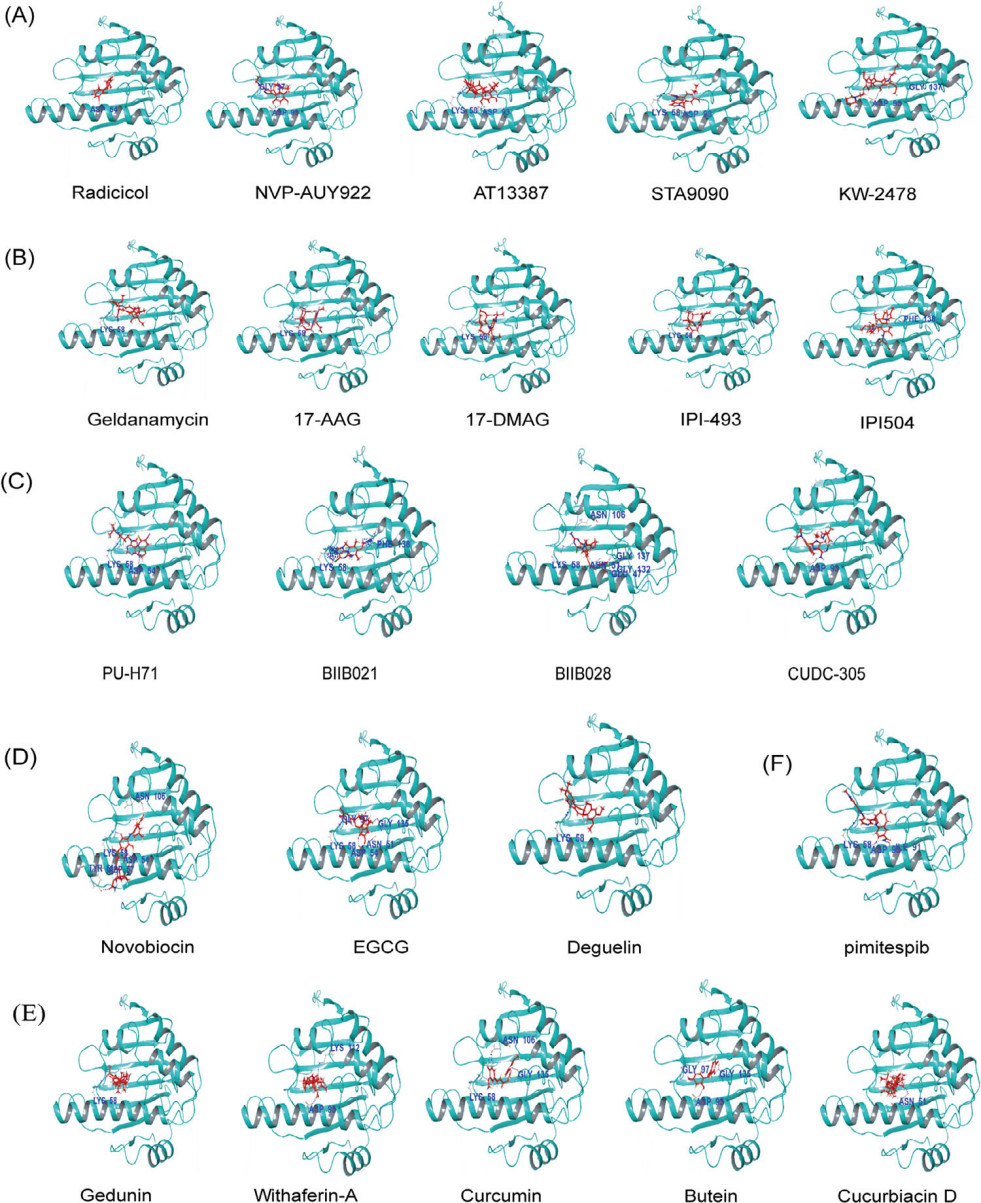
Chemical and 3D structures of representative inhibitors of HSP90. (A) Resorcinol compounds. (B) Benzoquinone compounds. (C) Purine‐based compounds. (D) CTD compounds. (E) PPI compounds. (F) Pimitespib.

HSP90 inhibitors are classified based on their mechanism of action as NTD or CTD inhibitors. The former, which are most commonly used, competitively bind to the ATP pocket on the NTD of HSP90. In contrast, the latter can block the dimerization of HSP90 and have a more flexible binding site compared with the NTD inhibitors.^82^ Furthermore, HSP90 inhibitors can be further divided into natural and synthetic inhibitors based on their source. Natural HSP90 inhibitors are extracted from plants and microorganisms. Based on their chemical structure, synthetic HSP90 inhibitors fall into different classes such as resorcinol, benzoquinone, purine‐based, CTD, and protein–protein interaction (PPI) compounds (Figures 4A–E). Furthermore, pimitespib (Figure 4F), an inhibitor with a distinct structure from the four aforementioned types, has received approval in Japan.^165^

### 8.1 Resorcinol compounds

Radicicol, a naturally compound extracted from the fungus Monosporium bonorden, exhibits strong inhibitory effects on HSP90 chaperone activity.^166^, ^167^ Radicicol demonstrates a robust binding affinity to NTD ATP pocket of HSP90, approximately 50 times stronger than GM.^167^ While radicicol exerts notable antitumor activity in vitro, its effectiveness against tumors in vivo is limited, potentially due to its conversion into inactive metabolites within the body.^168^ As a result, researchers have developed radicicol derivatives centered on the resorcinol core structure of these compounds. These derivatives have shown promising biological activity both in vitro and in vivo, as demonstrated through structure‐activity relationship analysis.^169^, ^170^ Further information regarding this type of inhibitor in clinical trials is outlined in Table 1.

**TABLE 1:** Clinical information of heat shock protein 90‐targeting resorcinol agents to inhibit ATPase cycle (NTD).^a^

Drug	MW (g/mol)	Phase	Status	Disease	Key endpoints	NCT
NVP‐AUY922	465.50	II	T	Pancreatic adenocarcinoma	DCR, ORR, PFS, OS, SDD	NCT01484860
				Metastatic disease		
				Myeloproliferative neoplasm	ORR	NCT01668173
			C	Non‐small cell lung cancer	ORR, OS, PFS	NCT01854034
				Non‐small cell lung cancer (ALK‐positive)	ORR, PFS, DCR	NCT01752400
NVP‐AUY922 + cetuximab	465.50 2756.23	I	C	Recurrent colon cancer	DLT, PRR, OS	NCT01294826
				Recurrent rectal cancer		
				Stage IV colon cancer		
				Stage IV rectal cancer		
				Colon adenocarcinoma		
				Rectal adenocarcinoma		
AUY922	465.50	I/II	C	Breast cancer	SD, efficacy, PK, PD	NCT00526045
				Hematological neoplasm		
AT13387	409.52	I	C	Solid tumor	Safety, tolerability, MTD, PK, PD	NCT01246102
				Breast cancer		
AT13387 + crizotinib	409.52 450.34	I/II	C	Non‐small cell lung cancer	DLT, ORR,	NCT01712217
AT13387 + imatinib	409.52 493.60	II	C	Gastrointestinal stromal tumor	Tumor size, PFS, OR, PK	NCT01294202
AT13387 + olaparib	409.52 434.46	I	C	Metastatic malignant solid neoplasm	MTD, DLT	NCT02898207
				Metastatic primary peritoneal serous adenocarcinoma		
				Metastatic triple‐negative breast carcinoma		
				Platinum‐resistant fallopian tube carcinoma		
				Platinum‐resistant ovarian carcinoma		
STA9090	364.40	I	C	Hepatocellular carcinoma	Phase 2 dose, AEs, TTP, PFS, OS	NCT01665937
		II		Ocular melanoma	PFS, DCR, ORR	NCT01200238
				Breast cancer	ORR	NCT01273896
				Colon and rectal cancer	ORR	NCT01111838
			T	Melanoma	6‐month PFR, BOR, OS	NCT01551693
			C	Esophagogastric cancer	Efficacy, PFS	NCT01167114
				Small cell lung cancer	8‐Week PFR, ORR, PFS, OS	NCT01173523
STA9090 + bortezomib + dexamethasone	364.40 384.24 392.47	I	C	Multiple myeloma	MTD	NCT01485835
STA9090 + paclitaxel + trastuzumab + pertuzumab	364.40 853.92 298.26 277.40	I	C	HER2‐positive breast cancer	MTD, ORR	NCT02060253
				Male breast cancer		
				Recurrent breast cancer		
STA9090 + docetaxel	364.40 807.88	I	C	Solid tumor malignancy	Safety, PK	NCT01183364
STA9090 + crizotinib	364.40 450.34	I	C	Advanced lung cancer	MTD, efficacy, OS, safety profile	NCT01579994
STA9090 + capecitabine	364.40 359.35	I	C	Rectal cancer	Tumor response, disease progression	NCT01554969
KW‐2478	574.66	I	C	Multiple myeloma Chronic lymphocytic leukemia B‐NHL	AEs, PK, PD, RP2D	NCT00457782
KW‐2478+ Bortezomib	574.66 384.24	I/II	C	Multiple myeloma	ORR, PK	NCT01063907

Abbreviations: AEs, adverse events; B‐NHL, B‐cell Non‐Hodgkin's Lymphoma; BOR, best overall response; C, completed; CR, complete response; DCR, disease control rate; DLT, incidence of dose limiting toxicity; DLT, dose limiting toxicities; MTD, maximum tolerated dose; MW, molecular weight; NTD, amino‐terminal domain; NCT, number of clinical trial; ORR, objective response rate; OS, overall survival; PFS, progression‐free survival; PR, partial response; PRR, patient response rate; PK, pharmacokinetics; PD, pharmacodynamics; PFR, progression‐free rate; RP2D, recommended phase II dose; SD, safe dose; SDD, stable disease duration; T, terminated; TTP, median time to progression.

^a^ The data were obtained from https://www.clinicaltrials.gov/.

NVP‐AUY922 is an exemplary derivative that has shown remarkable anticancer activity at the cellular level. When combined with doxorubicin, it significantly triggers apoptosis in the breast cancer cell line MCF‐7 and concurrently reduces VEGF expression.^171^ Notably, NVP‐AUY922 is among the extensively studied HSP90 inhibitors, exhibiting promising results in phase II clinical trials for various cancer types (registration numbers. NCT01484860 and NCT01854034).^172^, ^173^

AT13387, also called onalespib, is a second‐generation nonansamycin and fragment‐derived HSP90 inhibitor that effectively inhibits cell proliferation and survival in nasopharyngeal carcinoma (NPC), induces apoptosis of NPC cells by downregulating HSP90‐related substrate proteins such as EGFR, Akt, and cyclin‐dependent kinase 4 (CDK4), and inhibits tumor growth in xenograft models.^174^, ^175^, ^176^ Moreover, a phase II clinical trial of AT13387 combined with imatinib in gastrointestinal stromal tumor (GIST) has completed (No: NCT01294202). While it has exhibited well tolerance, its antitumor activity has proven to be limited.^177^

STA9090 is a groundbreaking catechol triazolone HSP90 inhibitor known for its effective reduction of ocular and liver toxicity, a distinctive feature attributed to its unique structure lacking benzoquinone. Furthermore, it has demonstrated superior safety and selectivity compared with other HSP90 inhibitors, making a remarkable advancement in the field.^178^ Studies have shown that STA9090 exhibits notable ability to inhibit tumor cell proliferation, with a half‐maximal inhibitory concentration of less than 1 μM/L, making it highly clinically applicable.^178^, ^179^, ^180^ In a phase I trial (No: NCT02060253) involving patients with HER2‐positive metastatic breast cancer, the combination of STA9090 with low concentrations of paclitaxel and trastuzumab has yielded promising results. Additionally, pertuzumab, an inhibitor of HER2, has effectively reduced resistance to single‐drug treatments, further enhancing its efficacy.^181^, ^182^ In addition, a phase I clinical trial of STA9090 (No: NCT01579994), which evaluated the combination of STA9090 and crizotinib for the treatment of advanced lung cancer, has been completed, demonstrating a superior antitumor effect compared with the use of either agent alone.^183^

KW‐2478, a nonansamycin and nonpurine compound, exhibits substantial anticancer potential through its targeted action on Hsp90's N‐terminal domain, disrupting its chaperone function and potentially eliminating NSCLC cells.^184^, ^185^ Nevertheless, a notable limitation in researching KW‐2478 is the absence of a comprehensive crystal structure involving HSP90 NTD‐KW‐2478, impeding further structural refinements and a deeper understanding of KW‐2478′s molecular mechanisms.^184^ Zeng and colleagues’^186^ study highlights KW‐2478′s capability to inhibit CML cell growth and induce apoptosis by inhibiting HSP90α, impacting the BCR/ABL and MAPK pathways. It also suggests promise for tyrosine kinase inhibitor (TKI)‐resistant and intolerant patients, pending dosage optimization. On the other hand, Zhao et al.’s^187^ research reveals that KW‐2478 lacks antiviral activity against porcine deltacoronavirus (PDCoV), while HSP90 inhibitors like 17‐AAG and VER‐82576 show potential in reducing PDCoV‐induced proinflammatory cytokines, positioning them as prospective candidates for PDCoV treatment. Furthermore, a phase I/II study (No: NCT01063907) evaluating KW‐2478 in combination with bortezomib demonstrated favorable safety profiles and initial clinical activity. This study indicated that KW‐2478, in conjunction with bortezomib, was well‐tolerated by patients with relapsed/refractory multiple myeloma, with no significant overlapping toxicity.^188^

### 8.2 Benzoquinone compounds

GM was the pioneering HSP90 NTD inhibitor to be discovered.^189^ Falling under the category of benzoquinone ansamycin antibiotics, GM has exhibited notable antitumor activity in vitro.^190^, ^191^ Initially, it was thought that GM directly targeted TYKs to exert its antitumor activity. However, subsequent work revealed that these antitumor effects are mediated by competitively combining to the ATP pocket within HSP90. This binding causes a conformational change that disrupts the formation of super‐complexes involving HSP90, co‐chaperones and client proteins. As a result, client proteins are irreversibly degraded, effectively blocking tumor‐dependent growth signaling pathways.^192^, ^193^ Despite its promising antitumor activity, the presence of benzoquinones in GM's structure causes severe hepatotoxicity and low solubility, thereby limiting its clinical application.^194^ Fortunately, derivatives with reduced toxicity and similar anticancer effects to GM have been discovered (Table 2).

**TABLE 2:** Clinical information of heat shock protein 90‐targeting benzoquinone agents to inhibit ATPase cycle (NTD).^a^

Drug	MW (g/mol)	Phase	Status	Disease	Key endpoints	NCT
17‐AAG + gemcitabine hydrochloride	585.69 299.66	II	C	Pancreatic adenocarcinoma	6‐month SR, OS, TtDP	NCT00577889
				Recurrent pancreatic cancer		
				Stage IV pancreatic cancer		
17‐AAG + rituximab	585.69 144,544.40	I	T	B cell chronic lymphocytic leukemia	MTD, MEPD	NCT00098488
				Prolymphocytic leukemia		
				Refractory chronic lymphocytic leukemia		
17‐DMAG	616.75	I	C	Large cell lymphoma	MTD, RP2D	NCT00089271
				Anaplastic large cell lymphoma		
				Angioimmunoblastic T cell lymphoma		
IPI‐493(17‐AG)	545.63	I	T	Hematological malignancy	EoSE, EoEE	NCT01193491
				Advanced malignancy	MTD, RP2D	NCT00724425
IPI504	624.20	II	C	Prostate cancer	Safety, Tolerability	NCT00564928
		I		Solid tumor	MTD, PK, CR, PR	NCT00606814

Abbreviations: C, completed; CR, complete response; EoSE, evaluation of safety endpoints; EoEE, evaluation of efficacy endpoints; MEPD, minimally effective pharmacological dose; MTD, maximum tolerated dose; MW, molecular weight; NTD, amino‐terminal domain; NCT, number of clinical trial; OS, overall survival; PR, partial response; PK, pharmacokinetics; RP2D, recommended phase II dose; SR, survival rate; T, terminated; TtDP, time to disease progression.

^a^ The data were obtained from https://www.clinicaltrials.gov/.

One of these derivatives, 17‐allylamine‐17‐demethoxygeldanamycin (17‐AAG), not only retains the GM's inhibitory activity against HSP90 but also exhibits decreased hepatotoxicity.^195^ Experimental results have highlighted 17‐AAG's capacity to selectively target tumor tissues with elevated HSP90 expression. Upon binding to HSP90, the drug induces tumor cell apoptosis even at lower concentrations. This phenomenon may be attributed to the abnormal activation of HSP90 in tumor cells, while it remains inactive in healthy cells, or possibly due to 17‐AAG's higher affinity toward HSP90 in tumor cells. Due to these antitumor advantages, 17‐AAG represents the first generation of successfully developed GM derivatives and the first HSP90 inhibitor to undergo worldwide clinical researches.^196^, ^197^, ^198^, ^199^ However, despite its potential, a phase II trial involving gemcitabine and 17‐AAG (No: NCT00577889) revealed unsatisfactory results in treating pancreatic cancer compared with gemcitabine alone. Specifically, targeting checkpoint kinase 1 (Chk1) by inhibiting HSP90 with 17‐AAG did not achieve the desired outcomes for pancreatic cancer treatment.^200^ To achieve better therapeutic effects, 17‐AAG is currently combined with other target anticancer agents. For example, the combination of 17‐AAG with paclitaxel synergistically induces tumor cell apoptosis and has shown significant antitumor effects in anaplastic thyroid carcinoma, NSCLC and bladder cancer.^201^, ^202^ However, 17‐AAG has a large molecular weight, an unstable structure, poor solubility, and is easily metabolized by liver enzymes, leading to low bioavailability.^196^

To address these limitations, several benzoquinone compounds such as IPI‐493, IPI‐504 and 17‐DMAG have been synthesized. These compounds are NTD HSP90 inhibitors and share a similar mechanism with 17‐AAG.^203^, ^204^, ^205^ Compared with GM, IPI‐493 exhibits enhanced bioactivity (with an EC50 of 34 nmol/L) and has demonstrated significant anti‐GIST effects while decreasing toxicity to some extent.^203^ Studies have revealed that when used in combination with TKIs like imatinib or sunitinib, IPI‐493 exerts a more potent antitumor effect than when used alone.^203^, ^206^ Furthermore, the combination of various HSP90 inhibitors with TKIs shows promising potential in overcoming resistance to imatinib or sunitinib in GIST models.^203^, ^207^, ^208^ However, phase I trial of IPI‐493 (No: NCT01193491) for hematological malignancy was terminated due to safety reasons.

Scaltriti et al.^209^ showed that IPI‐504 exhibits a promising antitumor effect in primary or acquired drug resistance models of HER2‐positive breast cancer caused by inactivation of the tumor suppressor PTEN or PI3K downregulation.^209^, ^210^ Antitumor effects of combination of IPI‐504 and trastuzumab have been demonstrated in advanced or metastatic HER2^+^ breast cancer patients in a phase II trial (No: NCT00817362).^211^ The standalone phase II trial of IPI‐504 (No: NCT00564928) for prostate neoplasms concluded in 2012; however, it exhibited limited efficacy and was associated with unacceptable toxicity in some patients. On the other hand, the phase II trial of IPI‐504 (No: NCT01362400) demonstrated clinical activity in patients with NSCLC, particularly among those with ALK rearrangements. Unfortunately, the phase III trial of IPI‐504 (No: NCT00688766) in GIST was terminated in 2012 due to safety concerns associated with hepatotoxicity.^212^, ^213^

### 8.3 Purine‐based compounds

PU3 was the pioneering HSP90 NTD inhibitor designed based on the purine structure. This compound contains the same 6‐amino‐purine ring as ATP and competitively binds to the NTD of HSP90 with ATP.^214^, ^215^ Using purine as the core scaffold, researchers have carried out structural optimizations to create PU3 analogs, such as PU24FCl and BIIB021 (Table 3).

**TABLE 3:** Clinical information of heat shock protein 90‐targeting purine‐based agents to inhibit ATPase cycle (NTD).^a^

Drug	MW (g/mol)	Phase	Status	Disease	Key endpoints	NCT No.
PU‐H71	512.37	I	T	Solid tumor lymphoma	DLTs, AEs, MTD	NCT01581541
			C	Metastatic breast cancer	MTD	NCT03166085
BIIB021	318.76	II	C	Advanced solid tumor	Safety, tolerability, PK	NCT01017198
				GIST	Safety, changes in FDG‐PET imaging	NCT00618319
BIIB028	465.83	I	C	Advanced solid tumor	Safety, tolerability, PK, PD	NCT00725933
CUDC‐305 (Debio 0932)	442.58	I	T	Non‐small cell lung cancer	DLTs, AEs	NCT01714037
CUDC‐305 (Debio 0932)	442.58	I	C	Solid tumors Lymphoma	DLTs, AEs, safety, efficacy	NCT01168752

Abbreviations: AEs, adverse events; C, completed; DLTs, cycle 1 dose‐limiting toxicities; GIST, gastrointestinal stromal tumor; MW, molecular weight; MTD, maximum tolerated dose; NTD, amino‐terminal domain; NCT, number of clinical trial; PK, pharmacokinetics; PD, pharmacodynamics; T, terminated.

^a^ The data were obtained from https://www.clinicaltrials.gov/.

PU24FCl rapidly targets tumor tissue and accumulates, exhibiting a range of antitumor activities.^216^ Further structural modification results in the development of PU‐H71. Studies have shown that PU‐H71 triggers apoptosis of triple‐negative breast cancer (TNBC) cells and downregulates expression of key factors such as VEGF, EGFR and Akt, thus inhibiting tumor growth.^217^, ^218^, ^219^ A phase I clinical trial assessed the safety and feasibility of combining PU‐H71 with nab‐paclitaxel in patients with metastatic breast cancer, showing promising results (No: NCT03166085).^220^

BIIB021 is a synthetic, novel, and orally available HSP90 inhibitor commonly used for the clinical treatment of solid tumors.^221^, ^222^, ^223^ Possessing stable physical and chemical properties, BIIB021has been successfully assessed with a clinical phase II trial (No: NCT00618319) in GIST, yielding positive and effective outcomes. Furthermore, BIIB021 administration has been observed to induce apoptosis in esophageal squamous cell carcinoma cells by downregulating essential proteins, such as Akt and EGFR, which play a pivotal role in overcoming tumor cell resistance to radiotherapy.^221^, ^224^, ^225^ In thyroid carcinoma cells, BIIB021 exhibits synergistic activity with triptolide, inducing cytotoxicity and degradation of HSP90 clients.^226^ Three phase I clinical trials involving patients with advanced solid tumors (registered as NCT01017198, NCT00618735, and NCT00345189) have been successfully completed, demonstrating the efficacy of BIIB021 treatment without any reported ocular or pulmonary toxicities.^223^

BIIB028, a second‐generation HSP90 inhibitor and a prodrug of BIIB021, has undergone structural optimization, resulting in enhanced antitumor activity compared with BIIB021.^227^ Currently, BIIB028 is undergoing a phase I study involving patients with advanced solid tumors (No: NCT00725933); BIIB028 has been demonstrated good tolerability, indicating its potential for further exploration in other cancer types.

CUDC‐305, also known as Debio 0932, is a second‐generation oral HSP90 inhibitor.^228^ This compound exhibits the ability to inhibit a wide range of signaling pathways, including the PI3K/AKT and RAF/MEK/ERK pathways. Importantly, it has been demonstrated to induce apoptosis while inhibiting cell apoptosis in models of breast cancer and MV4‐11 acute myelogenous leukemia.^229^ Additionally, a separate study suggested that Debio 0932 may hold promise in the treatment of psoriasis, based on observations of psoriasis remission in a xenograft model.^230^ However, it is worth noting that Debio 0932 was terminated in phase II clinical trial due to the absence of a clear dose–effect relationship and limited clinical activity.^228^

### 8.4 CTD compounds

CTD compounds primarily consist of natural molecules derived from plants and microorganisms, and additional information regarding this type of inhibitor in clinical trials are summarized in Table 4.

**TABLE 4:** Clinical information of heat shock protein 90‐targeting CTD agents to inhibit dimerization of Hsp90.^a^

Drug	MW (g/mol)	Phase	Status	Disease	Key endpoints	NCT no.
Novobiocin	612.62	I	T	Osteomyelitis	AEs, cost of care, clinical failures	NCT02099240
EGCG	458.37	Not applicable	C	Acne vulgaris	Acne severity, standardized clinical photographs	NCT01687556
		I/II	R	COVID‐19; pneumonia; malignant neoplasm	AEs	NCT05758571
		Not applicable	Unknown^b^	Nasopharyngeal carcinoma	OS, RFS, EBV reactivation rates	NCT01744587
		Early I	R	Colon cancer	Change in methylation	NCT02891538
EGCG + clomiphene citrate + letrozole	458.37 268.74 285.30	I	C	Uterine fibroids	PK, Hepatic safety	NCT04177693

Abbreviations: AEs, adverse events; C, completed; COVID, coronavirus disease; EGCG, epigallocatechin gallate; MW, molecular weight; NCT, number of clinical trial; NTD, amino‐terminal domain; OS, overall survival; PK, pharmacokinetics; R, recruiting; RFS, recurrence‐free survival; T, terminated.

^a^ The data were obtained from https://www.clinicaltrials.gov/.

^b^ Study has passed its completion date and the status has not been verified in >2 years.

Novobiocin and coumarin A1 are naturally occurring amino coumarin antibiotics isolated from *Streptomyces* species. Unlike the HSP90 inhibitors discussed earlier, which primarily target the NTD, novobiocin exerts its potent effects by primarily targeting the second ATP‐binding site located in CTD of HSP90. This interference disrupts the dimerization of HSP90, leading to the induced degradation of various client proteins, including RAF‐1, EGFR, and mutant p53, through the ubiquitin/proteasome pathway.^231^, ^232^

Epigallocatechin gallate (EGCG), the primary constituent of replenix, exerts its effects by inducing conformational changes in HSP90. This, in turn, interferes with the chaperone function of HSP90 and leads to a subsequent reduction in the expression of various HSP90 client proteins, such as Raf‐1, ErbB2, p‐ERK, p‐Akt, and Bcl‐2.^233^, ^234^, ^235^ Clinical trials of EGCG are currently ongoing (No: NCT02580279 and NCT02891538).^236^

Deguelin, classified as a flavonoid compound, exerts its effects by binding to the ATP pocket located in CTD of HSP90. This binding results in notable antiangiogenic, apoptosis‐inducing, and antiproliferative properties both in vitro and in vivo.^237^ Chen et al.^238^ demonstrated that deguelin hampers the proliferation of colorectal cancer cells by triggering apoptosis, primarily through the activation of the p38 MAPK signaling pathway. However, it has been associated with the induction of neurodegenerative diseases such as Parkinson's disease.^239^ To overcome this undesired side effect, scientists have developed and modified an analog of deguelin known as L80. L80 not only induces apoptosis in tumor cells but also circumvents the risk of causing neurodegenerative diseases, addressing the concern associated with the original compound. Cho et al.^240^ revealed that L80 effectively suppresses cell proliferation in TNBC cells while simultaneously inhibiting multiple signaling pathways, including AKT/MEK/ERK/JAK2/STAT3, within these cancer cells. Importantly, L80 does not induce cytotoxicity in normal cells, suggesting its potential as a targeted therapy for TNBC while sparing healthy cells.

The novel compound NCT‐50, an analog of novobiocin–deguelin, has demonstrated a remarkable capability in disrupting HSP90 function by directly binding to the ATP‐binding pocket of CTD of HSP90. As a result, NCT‐50 effectively downregulates the expression and activity of numerous HSP90 client proteins, including HIF‐1α, highlighting its promising potential as an anticancer drug targeting HSP90.^241^

NCT‐547, developed as a lead‐optimized derivative of L80, exhibits significant activity in HER2‐positive breast cancer. It functions by promoting the degradation of full‐length HER2 and truncated p95HER2 while reducing the heterodimerization of HER2 family members. These actions contribute to its effectiveness in targeting HER2‐related pathways in breast cancer.^242^

NCT‐58, a novel CTD HSP90 inhibitor, was rationally synthesized using O‐substituted analogs of the B‐ and C‐ring truncated scaffold of deguelin. This compound effectively suppresses cell proliferation and angiogenesis in a mouse xenograft model resistant to trastuzumab treatment. Remarkably, NCT‐58 achieves these effects without inducing a heat shock reaction (HSR), making it a promising candidate for overcoming trastuzumab resistance in breast cancer.^243^

NCT‐80, derived from a B‐ and C‐ring truncation of deguelin, effectively interferes with the interaction between HSP90 and STAT3. This interference results in reduced protein stability of STAT3 and inhibits STAT3‐mediated activation of the Wnt signaling pathway, demonstrating a potent antitumor effect in NSCLC.^244^

SL‐145 is an innovative C‐terminal HSP90 inhibitor derived from a C‐ring truncated deguelin derivative. It has demonstrated the ability to induce apoptosis in TNBC cells by effectively suppressing Akt, MEK/ERK, and JAK2/STAT3 signaling pathways. Importantly, SL‐145 achieves these effects without triggering the HSR.^245^

Zhang et al.^246^ demonstrated that penisuloxazin A reverses the epithelial‐mesenchymal transformation of breast cancer cells and enhances the cytotoxicity of natural killer cells against breast cancer cells. Dai et al.^247^ showed that penicisulfuranol A inhibits multiple HSP90 client proteins, induces apoptosis and inhibits xenograft tumor growth of HCT116 cells both in vitro and in vivo without inducing HSP70.

Cisplatin, a commonly used chemotherapy drug for various types of solid tumor, does not affect the formation of the HSP90–P23 complex, but suppresses formation of the HSP90–HSP70 complex.^248^, ^249^ It can inhibit the function of HSP90 by acting on the CTD of HSP90, thus inducing tumor cell apoptosis.^248^ The cisplatin derivative LA‐12 induces degradation of classical client proteins of HSP90 such as mutant p53. Compared with cisplatin, LA‐12 has a higher affinity for HSP90 and kills cisplatin‐resistant cancer cell lines.^250^ These results suggest that CTD inhibitors of HSP90 are promising anticancer agents worthy of further study.

### 8.5 PPI compounds

Inhibitors targeting the NTD and CTD of HSP90 represent an active area of development. However, the complexity and specificity of protein spatial structure make it challenging to directly inhibit HSP90, forcing researchers to explore alternative strategies to overcome these obstacles.^46^ In the dynamic and coordinated cycle of HSP90 chaperone activity, various co‐chaperones, including CDC37, P23, HOP, and AHA1, collaborate to facilitate the correct folding of numerous proteins, shielding them from undesired degradation.^71^, ^251^, ^252^ This insight suggests a potentially effective therapeutic approach, which involves indirectly inhibiting HSP90 function by interfering with or disrupting the interaction between HSP90 and its co‐chaperones.^160^ One potential approach is the development of PPI compounds, which represent a novel type of HSP90 inhibitor. Details regarding this type of inhibitors in clinical trials are summarized in Table 5.

**TABLE 5:** Clinical information of heat shock protein 90‐targeting protein–protein interaction (PPI) agents.^a^

Drug	MW (g/mol)	Mechanism	Phase	Status	Disease	Key endpoints	NCT no.
Withaferin‐A + DOXIL	470.60 543.53	Inhibiting PPI (HSP90–CDC37)	I/II	Not yet recruiting	Recurrent ovarian cancer	PFS, ORR, AEs	NCT05610735
Withaferin‐A	470.60	Inhibiting PPI (HSP90–CDC37)	Not applicable	C	Skin abnormality	Changes in appearance of facial redness or pigmentation	NCT04872946
Cucurbitacin D	516.67	Inhibiting PPI (HSP90–CDC37/P23)	III	C	Otitis media with effusion	Resolution of tympanic effusion Complete auditory recovery	NCT02858388
Curcumin	368.38	Inhibiting PPI	Not applicable	C	Prostate cancer	AEs, DTI	NCT03211104
			II	R	Pediatric acute lymphoblastic leukemia	Safety, efficacy	NCT05045443
			II	C	Head and neck cancer	Muscle mass, BMI, serum NF‐kB level	NCT04208334
					Advanced head and neck cancer	Muscle mass, BMI, serum NF‐kB level	
Curcumin + piperine	368.38 285.34	Inhibiting PPI	II	R	Prostate cancer; multiple myeloma	PFS	NCT04731844
					Smoldering multiple myeloma		
Curcumin	368.38	Inhibiting PPI	I	Active, not recruiting	Breast cancer	Tumor proliferation rate, AEs	NCT03980509
				R	Colon cancer	Safety, tolerability	NCT01294072
Curcumin + bioperine	368.38 285.34	Inhibiting PPI	Not applicable	C	Multiple myeloma	PBMC, NF‐kB	NCT00113841
Curcumin + gemcitabine	368.38 263.20	Inhibiting PPI	II	C	Pancreatic cancer	Response rate, survival, TTP	NCT00192842

Abbreviations: AEs, adverse events; BMI, body mass index; C, completed; DTI, duration of treatment interruption; MW, molecular weight; NCT, number of clinical trial; ORR, objective response rate; PFS, progression‐free survival; PPI, protein–protein interaction; PBMC, peripheral blood mononuclear cells; R, recruiting; TTP, time to tumor progression.

^a^ The data were obtained from https://www.clinicaltrials.gov.

Gedunin preferentially binds to P23, competitively blocking the normal binding of P23 to HSP90, which leads to degradation of client proteins such as HER2 by the ubiquitination/proteasome pathway.^253^ Gedunin has been used in the treatment of malaria and other infectious disease.^254^ Similarly, tripterine, while not a specific HSP90 inhibitor, effectively binds to cysteine residues on CDC37, disrupting the PPI between HSP90 and CDC37. This disruption leads to the instability and subsequent degradation of several HSP90 client proteins, including Akt and CDK4.^255^ Researchers such as Zuo et al.^256^ have observed that tripterine inactivates PI3K/AKT and JNK pathways, promoting the degradation of client proteins in MDA‐MB‐231 cells. Another compound, Withaferin‐A (WA), a major component of Withania somnifera, is also capable of interfering with the interaction between HSP90 and CDC37.^257^ Numerous studies have highlighted the potent antitumor effects of WA in cell and animal models of TNBC.^258^, ^259^, ^260^

Curcumin, a polyphenol first isolated from the root of herbaceous *Curcuma longa* in 1815, is well‐known for its potent antitumor and anti‐inflammatory activity.^261^ Jung et al.^262^ observed that curcumin induces the degradation of ErbB2 and Lee et al.^263^ showed that curcumin increases degradation of HSP90 mRNA. The efficacy of curcumin in treating head and neck neoplasms has been investigated through successful phase II clinical trials (No: NCT04208334 and NCT01160302), demonstrating that curcumin treatment is safe and well‐tolerated. Furthermore, patients with acute lymphoblastic leukemia are currently being recruited for the evaluation of curcumin In a phase II clinical trial (No: NCT05045443).

Fan et al.^264^ conducted a study revealing the remarkable properties of CO818, a curcumin derivative, in its interaction with and inhibition of the ATPase activity of HSP90. Similarly, Abdelmoaty et al.^265^ demonstrated that CO818 effectively induces the degradation of HSP90 client proteins, including RAS, RAF, MEK, ERK, and AKT. This inhibition directly impacts the RAS/RAF/MEK/ERK and PI3K/AKT pathways. Interestingly, CO818 achieves its effects by impeding the binding between HSP90 and its clients, while leaving their transcription unaffected. Consequently, the HSP90 clients are degraded through the proteasome pathway rather than the lysosome pathway. These findings present CO818 as a promising candidate for targeted cancer therapy.^265^

3,4,2′,4′‐Tetrahydroxychalcone (butein), a chacolnoid isolated from various plant sources, induces the degradation of several HSP90 clients, including MMP2, MMP9, EGFR, Akt, STAT3, and VEGF in various cancer cell lines.^266^, ^267^, ^268^ Paclitaxel, a natural anticancer drug, interferes with mitosis and inhibits tumor cell proliferation by stabilizing microtubules and preventing microtubule depolymerization. It has notable antitumor effects in ovarian, breast, and uterine cancer.^269^, ^270^ Although paclitaxel binds to HSP90 and has an antiproliferative effect, the detailed mechanism of action remains unclear.

Hall et al.^271^ showed that the compound cucurbitacin D, derived from *Cucurbita texana*, inhibits the maturation of HSP90 clients without inducing the HSR. This effect was achieved by disrupting the interaction between HSP90 and its co‐chaperones, CDC37 and P23. Interestingly, another compound called 3‐epi‐isocucurbitacin D did not impact the HSP90 co‐chaperone interaction but still led to client protein degradation through an alternative mechanism, disrupting HSP90 client maturation in a different way.

Furthermore, taxifolin, a natural phytochemical, was also found to be a significant HSP90 inhibitor. Taxifolin binds to HSP90 and disrupts the interface residues of the HSP90 and CDC37 complex, playing a crucial role in inhibiting HSP90 and offering a novel approach for cancer treatment. These findings highlight the potential of these compounds as promising candidates for further exploration in cancer research.^272^

In recent research, a promising small molecule inhibitor called DDO‐5936 has been identified as a potential HSP90 disruptor. This inhibitor effectively interferes with the PPI between HSP90 and CDC37, both in laboratory settings (in vitro) and in living organisms (in vivo), specifically in colorectal cancer.^159^ Furthermore, Chen et al.^273^ conducted a study on another inhibitor known as DCZ3112, which also targets the HSP90–CDC37 PPI. This compound has shown significant potential as a targeted approach against HER2‐positive breast cancer, especially in cases where patients have developed acquired resistance to anti‐HER2 antibodies. Combining DCZ3112 with anti‐HER2 antibodies could offer a promising strategy for combating this type of breast cancer and overcoming treatment resistance.

In summary, PPI inhibitors targeting HSP90 have shown the ability to downregulate clients without affecting the ATPase activity of HSP90, making them promising candidates for clinical application. However, challenges remain in the development of PPI inhibitors, particularly in improving binding activity to HSP90 and effectively anchoring the flat binding sites.^274^

### 8.6 Pimitespib

Pimitespib, marketed as Jeselhy®, stands as the sole HSP90 inhibitor to have gained approval in the Japanese market for the treatment of GIST in June 2022.^165^ This oral small molecule inhibitor is specifically designed to target HSP90 α and β isoforms and is currently in development by Taiho Pharmaceutical for treating various solid tumors, including GIST. A phase Ib clinical trial, registered as JPRN‐jRCT2031220179, was conducted as an open‐label study focused on dose‐finding and expansion. The results from this trial revealed that when pimitespib was administered at a dose of 160 mg in combination with nivolumab, it displayed manageable safety profiles and exhibited antitumor activity. Notably, this combination therapy showed particular effectiveness in patients with microsatellite‐stable colorectal cancer.^275^ Furthermore, in a randomized, double‐blind, placebo‐controlled phase III trial, registered as JPRN‐jRCT2080224033, involving 86 patients with advanced GIST refractory to standard TKIs, pimitespib demonstrated significant clinical benefits. The results indicated that pimitespib significantly improved progression‐free survival and overall survival after adjusting for cross‐over effects when compared with a placebo. Importantly, the treatment maintained an acceptable safety profile.^163^ However, it is essential to note that adverse events associated with this treatment, particularly diarrhea (occurring in 74.1% of cases) and decreased appetite, should not be overlooked.

In summary, these inhibitors mentioned above, ranging from small molecules to natural compounds, have shown promise in targeting HSP90 and its isoforms, offering new avenues for the treatment of various diseases, particularly cancer. While significant progress has been made in understanding the intricacies of HSP90 inhibition, further research is needed to optimize their development, improve their specificity, and minimize adverse effects. The dynamic landscape of HSP90 inhibitors continues to evolve, and their potential for personalized medicine and combination therapies holds great promise for the future.

## 9 CONCLUSION AND PROSPECTS

Given the close association of HSP90 with the pathogenesis of various diseases, particularly cancer, targeting this molecular chaperone to eliminate aberrant cells while protecting normal ones holds immense therapeutic potential. This review provides a comprehensive assessment of the multifaceted functions and characteristics of HSP90 as a molecular chaperone, its involvement in a wide array of diseases, and its intricate connections with cancer resistance. With its abundance and distinct structural characteristics, HSP90 has rightfully become a focal point of research and innovation, attracting considerable attention and investigation.^9^, ^276^, ^277^, ^278^

However, harnessing the potential of HSP90 is not without its challenges. One notable issue is the tendency of certain NTD HSP90 inhibitors to upregulate the expression of HSP70 and other HSPs, ultimately promoting cytoprotection and yielding less than satisfactory results. This dilemma has spurred the development of the CTD and PPI inhibitors, which show promise in avoiding this protective response. Furthermore, while HSP90 inhibitors have demonstrated remarkable therapeutic effects in mechanistic studies, clinical trials have not consistently yielded the expected outcomes. As of now, only one HSP90 inhibitor, pimitespib, has gained approval in Japan for GIST treatment. The lack of specificity in these inhibitors, largely due to the sequence identity among the four HSP90 isoforms, particularly the 85% similarity between HSP90α and HSP90β, remains a significant limitation.^9^, ^279^ Therefore, it is important to continue research on isoform‐selective inhibitors, with the aim of mitigating generalized HSP90 inhibition. For instance, Mishra et al.^280^, ^281^ analyzed differences between Hsp90α and Hsp90β in the ATP binding site, then used fluorescence assays to create highly selective inhibitors for Hsp90α and Hsp90β without raising Hsp90 levels, a significant advance in isoform‐selective inhibitors.

In addition, the complex regulatory role of HSF‐1 in HSR presents another avenue of exploration.^59^ HSP90 inhibitors, in combination with HSF‐1 inhibitors, could potentially offer a novel therapeutic strategy. Moreover, some researchers also believe that molecular dynamics studies of drug binding to HSP90 have a significant impact on drug efficacy. Yang et al.^282^ used molecular dynamics simulations to highlight van der Waals interactions as key forces in inhibitor‐HSP90 binding. Hydrogen bonding and hydrophobic interactions were also identified, with specific hotspots on HSP90. Chen et al.^283^ corroborated the role of these interactions in enhancing binding. Additionally, molecular dynamics simulations were used to evaluate the absorption, distribution, metabolism, excretion, and toxicity and physicochemical properties of potential anticancer molecules targeting HSP90.^284^, ^285^ While research on HSP90 binding kinetics is ongoing, it holds promise for improved clinical use of HSP90 inhibitors in the future.

In summary, the studies focused on HSP90 in cancer treatment present several feasibility and prospect. Firstly, given the intricate interactions involving HSP90, co‐chaperones, and client proteins, HSP90 inhibitors are designed to regulate key clients in cancer by disrupting their normal interactions. However, current research primarily centers on understanding the effects of inhibitors on individual cell death processes, with complexities such as chaperone‐mediated autophagy and necrosis. One of the key challenges in utilizing HSP90 as a therapeutic target is selecting the appropriate inhibitors that can either promote normal cell survival or inhibit overall tumor cell activity.^286^ Next, NTD inhibitors of HSP90 have the capacity to activate HSR and elevate HSP70 expression, providing robust cellular protection. Lower doses of HSP90 inhibitors can effectively trigger the HSR without causing widespread cytotoxicity. Therefore, determining the appropriate dosages of HSP90 inhibitors is a crucial aspect of cancer treatment. Despite these challenges, HSP90's role in addressing cancer remains highly promising. Third, targeting the indirect inhibition of HSP90 activity through the silencing of co‐chaperones, a mechanism commonly employed by natural inhibitors like celastrol and gedunin, presents an effective strategy to circumvent drug resistance. HSP90 client proteins are deeply involved in homeostasis processes, and the selectivity of HSP90 inhibitors against “tumor HSP90” offers significant advantages. Natural HSP90 inhibitors have contributed significantly to enhancing our understanding of the connection between HSP90 and cancer, as well as the identification and development of novel semi‐synthetic or fully synthetic HSP90 inhibitors. Although off‐target toxicity remains a substantial challenge with natural HSP90 inhibitors, progress is evident through the development of natural product‐based derivatives aimed at creating highly effective and less toxic HSP90 inhibitors.^287^ To fully realize this potential, further work is essential that are concentrated on designing highly specific inhibitors for the distinct Hsp90 isoforms, advancing the development of derivatives based on natural HSP90 inhibitors, optimizing dosages for these agents, and enhancing our understanding of their interactions with key co‐chaperones. The ongoing clinical trials are essential for evaluating the safety and efficacy of these inhibitors. Furthermore, emerging technologies like cryo‐electron microscopy (cryo‐EM) and advancements in structural biology are anticipated to unveil the finer details of HSP90–client interactions. This deeper understanding will enable the design of even more precise inhibitors.

In conclusion, understanding and work on HSP90 is far from being over. While the challenges in targeting HSP90 for cancer and disease treatment are evident, they are by no means insurmountable. The field represents high complexity, but also brimming with new possibilities. As we continue to unravel new understanding of HSP90 and its inhibitors, we inch closer to harnessing its therapeutic potential for the benefit of human health. This review serves as a stepping stone in this exciting scientific endeavor, opening doors to novel therapeutic strategies and offering hope for improved treatments in diverse disease contexts.

## AUTHOR CONTRIBUTIONS

G. H., H. W., and P. H. conceived and designed the study. H. W. wrote the manuscript. Y. Z. performed the literature review. Y. J., X. C., and T. N. constructed the figure the tables. A. C. revised and edited the manuscript. G. H. and P. H. revised the manuscript. All authors reviewed and approved the final manuscript.

## CONFLICT OF INTEREST STATEMENT

The authors declare no conflicts of interest.

## ETHICS STATEMENT

Not applicable.

## Data Availability

Not applicable.
